# Exploring the use of a digital visual collaboration platform in pediatric clinical teaching: medical students’ experiences and perceptions—cross-sectional study

**DOI:** 10.3389/fmed.2026.1818596

**Published:** 2026-04-28

**Authors:** Laila AlBishi

**Affiliations:** Department of Pediatric, University of Tabuk, Tabuk, Saudi Arabia

**Keywords:** clinical reasoning, digital tools, medical education, Miro app, pediatric module, student satisfaction, visual learning

## Abstract

**Background:**

Digital visual collaboration tools are increasingly used in clinical education to promote engagement, reasoning, and interactive learning. Miro, a digital workspace, supports student-centered, case-based learning. This study aimed to assess medical students’ satisfaction, perceptions, and experiences with Miro during a pediatric clinical module.

**Methods:**

A cross-sectional survey was conducted among fifth-year medical students at the Faculty of Medicine, University of Tabuk. Miro was implemented as a learning approach in small-group pediatric case-based sessions. The self-administered questionnaire included 29 items across six domains: perception of the learning method, satisfaction with the learning experience, experience with Miro as a tool, cognitive reasoning and analytical processes, self-directed learning, and perceived challenges. Descriptive and comparative analyses were performed, including gender-based comparisons using Mann–Whitney *U* and chi-square tests.

**Results:**

A total of 136 students (76 females, 60 males) participated. Overall, students reported positive perceptions and satisfaction with Miro, with mean scores exceeding 4.0 on most items. The highest-rated statements were “The contents and composition of the topic were suitable to the students’ level” (mean = 3.93 ± 1.00) and “I was able to learn how to build a hypothesis about the case scenario” (mean = 3.86 ± 0.90). Most students rated analytical and reasoning tasks as reasonable in difficulty. Gender analysis revealed significant differences in perceived barriers and educational value: males reported higher perceived engagement, whereas females more often cited vague goals and limited resources.

**Conclusion:**

Miro was positively perceived by students as a visual learning platform in pediatric clinical modules. While students of all genders found it beneficial, gender-based disparities in experience and perceived challenges underscore the need for inclusive instructional strategies and targeted support to optimize digital learning environments.

## 1 Introduction

The rapid adoption of digital tools in education has changed how students learn, collaborate, and interact with course content. This shift has led to an increased focus on student-centered learning, blended approaches, and the flipped classroom, enabling students and professionals to access clinical data, medical apps, and scientific journals directly in clinical and ward-based settings (1). As a result, recent umbrella reviews indicate that digital and emerging technologies in medical education improve learner engagement, knowledge acquisition, and overall effectiveness (2). Concurrently, medical students are increasingly collaborating and building communities by creating and sharing educational materials online through social media platforms (3). Despite these advances, the role of the facilitator in learner-centered models is often underdeveloped. Ideally, facilitators actively mentor students in critically assessing, synthesizing, and applying information from diverse sources—helping them navigate unfiltered or conflicting content, encourage reflective questioning, and support evidence-based decision-making. Nevertheless, in practice, facilitators’ involvement is sometimes limited, and learning effectiveness may decline when students encounter unfiltered or poorly validated information from multiple, often conflicting, sources (4). The integration of visual workspace technology allows learners to control their own learning and transforms the teacher’s role from provider of information to facilitator of knowledge (5), that integration has improved learning satisfaction (6). This type of approach typically occurs in asynchronous collaboration settings, where students and teachers can collaborate at different times (7).

Miro is a leading visual collaboration platform that enables real-time and asynchronous teamwork through a digital whiteboard. Since 2011, it has provided an infinite canvas for brainstorming, mind mapping, and team planning with features such as templates, sticky notes, and integrations with major productivity tools. In medical education, Miro supports student-centered learning by facilitating case discussions, collaborative diagramming, and connecting in-person and remote participants.

Studies show that visual workspace technologies have significantly transformed teaching and learning in medical disciplines, positioning medical education at the forefront of curricular integration (8, 9). As hybrid and digital modalities become more common, tools like Miro offer innovative ways to reinforce knowledge, foster teamwork, and simulate clinical reasoning in accessible formats (10). Constructivist learning theory states that knowledge is actively built through interaction, collaboration, and engagement with meaningful tasks rather than passively received (11, 12). Digital collaborative platforms such as Miro support this approach by allowing learners to externalize their thinking, solve problems together, and interact within shared visual environments. Features like real-time collaboration, visual mapping, and iterative discussion may enhance cognitive engagement and support the development of clinical reasoning. This alignment provides a conceptual framework for understanding how visual collaboration tools can shape students’ learning experiences and outcomes.

In light of these trends and potential benefits, this study aimed to assess fifth-year medical students’ experiences, perceptions, and satisfaction with the Miro app as a visual workspace for collaborative learning during their pediatric module rotation.

## 2 Materials and methods

### 2.1 Study design

This cross-sectional study was conducted between September and November 2025 and targeted fifth-year medical students enrolled in the pediatric clinical module at the Faculty of Medicine, University of Tabuk. The study was approved by the University of Tabuk Research Ethics Committee (Approval No. UT-685-307-2025; September 8, 2025). All procedures were conducted in accordance with the ethical standards set out in the Declaration of Helsinki.

### 2.2 Study setting and participants

Collaborative learning strategies, including problem-based learning (PBL), team-based learning (TBL), and case-based collaborative learning (CBCL), are formally integrated into the clinical-phase curriculum and constitute at least 30% of instructional activities. A total of 170 fifth-year medical students participated in the pediatric clinical module during the study period. All students were exposed to the Miro-based collaborative learning sessions. Only fully completed questionnaire responses were included in the final analysis to ensure data integrity. Partial or incomplete responses were excluded. During the pediatric module, the Miro application was implemented as a visual workspace tool to enhance interactive and collaborative learning. Students were divided into small groups of 6–8 members, consistent with active learning pedagogies such as PBL and TBL.

Before each session, students received a digital invitation containing:

- A clinical case scenario with open-ended guiding questions
- Access to a shared Miro board

Students were encouraged to consult external online resources to support clinical reasoning and decision-making. The facilitator had real-time access to each group’s board, enabling close observation of individual contributions. This allowed monitoring of how students approached the case, facilitated targeted interaction with each group, and enabled immediate feedback and encouragement.

### 2.3 Educational intervention: Miro-based collaborative learning model

The Miro application was implemented as a structured visual collaboration tool during the pediatric clinical module to support case-based small-group learning. The intervention was integrated within existing collaborative learning formats, including PBL and TBL. It was delivered across multiple scheduled module sessions during the study period. Students were organized into small groups of 6–8 participants to facilitate active engagement and peer interaction. Before each session, students received a digital invitation containing a clinical case scenario accompanied by open-ended guiding questions designed to stimulate analytical reasoning and hypothesis generation. The invitation also included access to a dedicated shared Miro board.

The Miro platform served as a cloud-based digital whiteboard, enabling synchronous collaboration. Its interactive features included handwritten input, keyboard-based text entry, drawing tools, concept mapping, flow diagram construction, and digital sticky notes. These tools allowed students to visually organize clinical data, identify relevant cues, construct differential diagnoses, and collaboratively develop logical reasoning pathways. Students were encouraged to consult external online resources during the sessions to promote self-directed learning and evidence-based reasoning. This approach aimed to foster autonomy while maintaining structured facilitation.

The course facilitator had real-time access to each group’s Miro workspace, enabling continuous observation of student contributions and reasoning processes. This allowed the facilitator to monitor individual participation, provide immediate formative feedback, clarify misconceptions, and encourage reflective discussion. The intervention was designed to enhance cognitive engagement, promote collaborative problem-solving, and scaffold the development of clinical reasoning skills through visual and interactive learning strategies. This approach was grounded in constructivist learning principles, where learners actively construct knowledge through interaction, collaboration, and engagement with clinical scenarios.

### 2.4 Data collection instrument

Data was collected using a structured, self-administered electronic questionnaire developed specifically for this study. The instrument consisted of 29 closed-ended items organized into six domains:

1. Perception of the learning method
2. Satisfaction with the learning experience
3. Experience with the Miro app as an educational tool
4. Cognitive reasoning and analytical processes
5. Self-directed learning and goal setting
6. Perceived challenges and barriers

Response formats included:

- A 5-point Likert scale where responses are recorded as a 5-point scale ranging from 1 (strongly disagree) to 5 (strongly agree).
- A 3-point difficulty scale (Easy, Reasonable, Difficult)
- Yes/No/Maybe responses

Demographic information included student gender, which was used to compare subgroups. The questionnaire was created in English and distributed via Google Forms to all eligible students. Before participating, students were provided with an overview of the study and an electronic informed consent statement. Participation was voluntary, and responses were collected anonymously without personal identifiers, which may reduce but not eliminate the risk of response bias, including social desirability bias. Internal consistency of the questionnaire was assessed using Cronbach’s alpha coefficient.

### 2.5 Data analysis

Data were exported from Google Forms into Microsoft Excel for initial cleaning and verification, then imported into IBM SPSS Statistics (version 25) for analysis. Descriptive statistics were used to summarize participant characteristics and response distributions. Frequencies and percentages were calculated for categorical variables, including gender and responses to three-point and binary items. For Likert-scale items, responses were numerically coded from 1 to 5. Means and standard deviations (SDs) were calculated for individual items. Composite domain scores were calculated by summing the responses to items within each domain (e.g., perception, satisfaction, and experience), with higher scores indicating more positive responses. To assess gender-based differences in student perceptions, satisfaction, and learning experiences, the Mann–Whitney *U* test was applied to compare Likert-scale variables (e.g., perception, satisfaction, and experience scores) between male and female students. The chi-square (*χ*^2^) test was used for categorical variables, including difficulty ratings (easy, reasonable, difficult) and Yes/No/Maybe responses. The effect sizes (*r*) were calculated from the standardized test statistic for Mann–Whitney *U* tests and interpreted as small (0.1), medium (0.3), or large (0.5) to assess the magnitude of differences. Where appropriate, 95% confidence intervals (CIs) were reported. A *p*-value < 0.05 was considered statistically significant. Because this is an exploratory educational study, adjustments for multiple comparisons were not applied, which is appropriate for hypothesis-generating analyses.

### 2.6 Ethical considerations

The study was approved by the University of Tabuk Research Ethics Committee (Approval No. UT-685-307-2025; September 8, 2025). All participants provided electronic informed consent before data collection. Participation was voluntary and anonymous, and all procedures complied with institutional and international ethical standards.

## 3 Results

A total of 170 fifth-year medical students were enrolled in the pediatric clinical module during the study period. Of these, 136 students completed the questionnaire in full and were included in the final analysis, yielding a response rate of 80.0%. All respondents had participated in the Miro-based small group learning activities during the module. Only fully completed questionnaires were retained for analysis to ensure data completeness and accuracy; incomplete responses were excluded. The questionnaire demonstrated good internal consistency, with a Cronbach’s alpha coefficient of 0.85 indicating strong reliability.

### 3.1 Perceptions and satisfaction with Miro in pediatric clinical teaching

Overall, students reported positive perceptions and satisfaction with the Miro-supported learning experience, as shown in Table 1. Mean scores exceeded 3.7 for all items, indicating generally favourable responses across domains. The highest-rated items were “The contents and composition of the topic were suitable to the students’ level” (mean = 3.93 ± 1.00) and “I was able to learn how to build a hypothesis (clinical reasoning) about the case scenario” (mean = 3.86 ± 0.90). Gender-based comparisons revealed statistically significant differences across several items (*p* < 0.05), with male students generally reporting higher levels of perceived engagement and educational value.

**Table 1:** Perceptions, satisfaction, and perceived educational value of the Miro platform, with gender-based comparisons.

Item	Mean	Standard deviation (SD)	Male mean (95% CI)	Female mean (95% CI)	*p*-value	Effect size (*r*)
A. Perception and satisfaction						
It is a more effective method of learning than traditional lectures	3.74	1.16	4.14 (3.93–4.34)	3.44 (3.15–3.73)	<0.05^*^	0.21
I am more motivated to learn in such an activity	3.82	1.06	4.20 (4.03–4.38)	3.52 (3.25–3.79)	<0.05^*^	0.26
The contents and composition of the topic were suitable for the students’ level	3.93	1.00	4.05 (3.83–4.27)	3.52 (3.24–3.79)	0.711	0.26
I was able to learn how to build a hypothesis (clinical reasoning) about the case scenario	3.86	0.90	3.98 (3.75–4.22)	3.88 (3.65–4.12)	<0.05^*^	0.18
I am satisfied with the assessment method	3.75	1.11	4.07 (3.88–4.26)	3.70 (3.48–3.92)	<0.05^*^	0.22
C. Perceived educational value of the Miro app						
The use of visual technology (Miro) has educational value	3.70	0.56	3.92 (3.84–3.99)	3.53 (3.38–3.68)	<0.05^*^	0.26
It helps to achieve the topic objectives more effectively	3.60	0.66	3.90 (3.82–3.98)	3.36 (3.19–3.54)	<0.05^*^	0.33
Makes teamwork more interesting	3.67	0.61	3.81 (3.68–3.95)	3.56 (3.41–3.71)	<0.05^*^	0.18
Keeps me motivated	3.71	0.60	3.93 (3.87–4.00)	3.53 (3.37–3.70)	<0.05^*^	0.24
I trained in logical thinking and judgment	3.59	0.65	3.86 (3.77–3.95)	3.38 (3.21–3.55)	<0.05^*^	0.30
My discussion capability and communication with the group have improved	3.64	0.63	3.88 (3.80–3.97)	3.45 (3.29–3.62)	<0.05^*^	0.26

SD, standard deviation; CI, confidence interval; Miro, cloud-based visual collaboration platform; *p*-value, probability value. Significance set at *p* < 0.05 (^*^). Effect size (*r*) calculated from the Mann–Whitney *U* test and interpreted as small (0.1), medium (0.3), and large (0.5).

### 3.2 Perceived educational value of Miro

Students reported positive perceptions regarding the educational value of Miro, as shown in Table 1. The use of visual technology (mean = 3.70 ± 0.56) and teamwork activities (mean = 3.67 ± 0.61) were rated particularly highly. Significant gender-based differences were observed for all six items in this domain, with male students consistently reporting higher mean scores (*p* < 0.05). Effect sizes ranged from small to moderate (*r* = 0.18–0.33), indicating varying magnitudes of gender-based differences.

### 3.3 Students’ perception of analytical and reasoning processes

Table 2A shows that the majority of students rated analytical and reasoning tasks as “reasonable” in difficulty, indicating a moderate level of perceived cognitive demand. Approximately 68% rated the combination of clue recognition and reasoning as reasonable, while 10–14% found it challenging. Gender analysis showed no statistically significant differences (*p* > 0.05).

**Table 2:** Students’ perceived cognitive difficulty and learning barriers with gender comparison.

A. Analytical and reasoning processes (difficulty scale)				
Item	Easy (%)	Reasonable (%)	Difficult (%)	*p*-value
Recognizing clues in the case scenario	15.4	69.9	14.7	0.564
Combining clues logically to develop clinical reasoning	18.4	72.8	8.8	0.676
Setting up a learning goal	27.2	63.2	9.6	0.226

B. Perceived barriers and challenges (yes/no/maybe responses)				
Item	Yes (%)	No (%)	Maybe (%)	*p*-value
The goal was vague and confusing	19.5	44.2	36.4	<0.05^*^
There is an unfair student evaluation	9.6	72.8	17.6	0.05
There are insufficient learning resources	11.8	68.4	19.9	<0.05^*^
The topic is rare and difficult to study	14.0	64.0	22.1	<0.05^*^

*p*-value, probability value; *p* < 0.05 (^*^); analyses performed using chi-square test.

### 3.4 Perceived barriers and challenges

Students reported several perceived learning barriers related to goal clarity, evaluation fairness, and the adequacy of learning resources, as shown in Table 2B. Female students were significantly more likely to report challenges, such as vague learning goals (19.5%), unfair evaluation (15.6%), and insufficient resources (19.9%), than male peers (*p* < 0.05). These findings indicate differences in reported experiences of instructional and contextual factors within the collaborative learning environment.

### 3.5 Self-directed learning and goal-setting tasks

**Figure 1:**
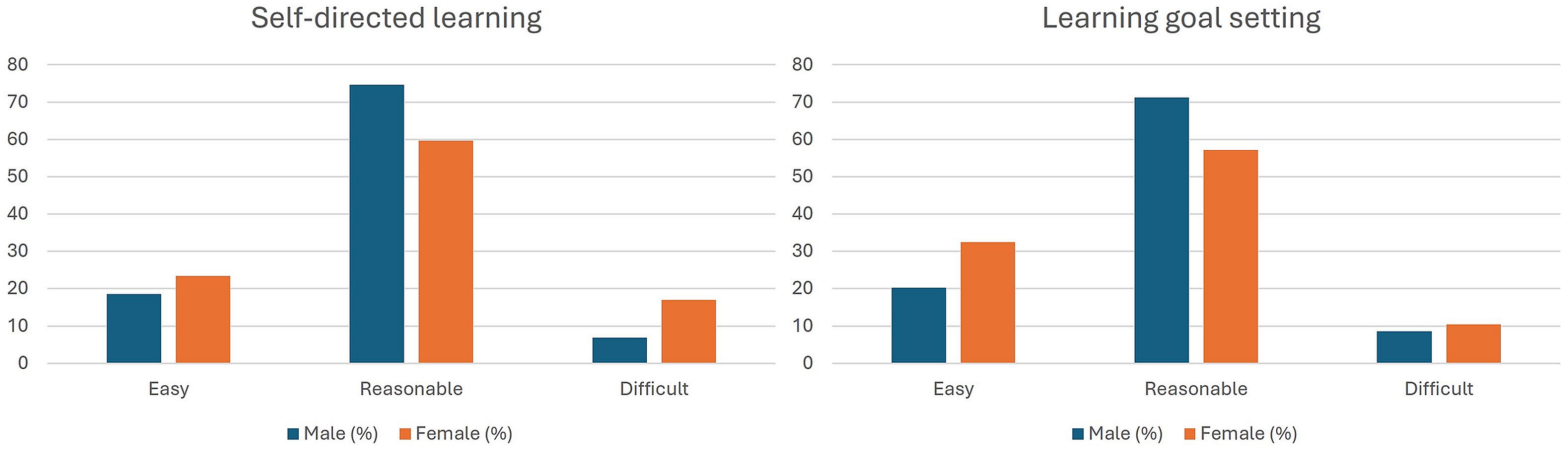
Gender-based distribution of perceived difficulty in self-directed learning and goal-setting tasks. Percentages represent responses within each gender group. The majority of students in both groups rated the tasks as “reasonable” in difficulty, with a slightly higher proportion of female students reporting “easy” responses and more females than males reporting “difficult” responses.

Gender-based perceptions of self-directed learning and goal-setting tasks are presented in Figure 1. Most students in both groups rated these tasks as “reasonable” in difficulty, indicating a moderate and manageable level of perceived challenge. Male students more frequently reported tasks as “reasonable” compared to female students, particularly in self-directed learning (74.6% vs. 59.7%) and goal setting (71.2% vs. 57.1%). In contrast, a higher proportion of female students rated tasks as “easy,” especially for goal setting (32.5% vs. 20.3%). Additionally, female students were more likely to report tasks as “difficult” compared to male students, particularly in self-directed learning (16.9% vs. 6.8%). Overall, these findings indicate broadly similar patterns across genders, with moderate differences in perceived ease and difficulty.

## 4 Discussion

The study found that fifth-year medical students generally viewed the Miro app positively as a visual learning tool, with no major gender differences in overall perceptions or satisfaction. This aligns with prior research on collaborative digital platforms in health professions education, which can boost engagement and promote advanced cognitive skills, such as clinical reasoning (13, 14). The notably high scores regarding content suitability and hypothesis generation indicate that students valued Miro’s visual, interactive approach for supporting problem-solving and self-directed learning. Framing these results within a constructivist learning approach, the findings highlight the benefits of active, collaborative engagement for motivation and strategy development (15). Based on these findings, educators could effectively incorporate visual collaboration tools such as Miro to foster active learning and critical thinking, provided that these tools align with instructional aims and are thoughtfully integrated (16).

The Miro platform may enable shared visual interaction and collaborative reasoning, supporting cognitive engagement by making clinical reasoning tasks manageable. Most participants rated clue recognition, logical reasoning, and goal setting as “reasonable” in difficulty, indicating that the instructional design supported complex cognitive tasks (17). Evidence shows that well-designed digital environments can reduce cognitive load and facilitate more efficient knowledge processing (18–20). The low proportion of students reporting tasks as “difficult” indicates the platform’s accessibility for information-rich, problem-based activities (21, 22). The absence of significant gender-based differences further supports the platform’s usability across different learner groups (23).

Key findings indicate that gender-based differences emerged mainly in perceived barriers and challenges. Female students more often expressed concerns about unclear learning goals, fairness in evaluation, sufficiency of resources, and task complexity. This aligns with the literature showing that female learners may focus more on the instructional context, especially during self-regulated or technology-mediated education (24, 25). Such differences may reflect broader sociocultural factors and learning preferences, with female students placing greater emphasis on the clarity of structured assessments (26, 27). These concerns might increase stress when expectations are vague. Differences in support or digital confidence may further affect resource use (28). To address these, instructors could make instructions, learning goals, and evaluation criteria more explicit, provide structured feedback, and offer tailored guidance. Regular check-ins can help all learners feel supported.

Gender-based differences in perceived educational value were also observed, with male students reporting higher perceived benefits across several domains. These results underline the importance of monitoring and addressing potential differences in confidence or perceptions of new technologies (29, 30). Educators should consider support strategies to ensure that all students can engage equally with digital tools, acknowledging possible variation in preferred learning approaches and experiences.

From a pedagogical perspective, these findings underscore the need for structured implementation when adopting digital learning tools. Orientation and scaffolded training can support students with varying levels of digital familiarity, while clear learning objectives and evaluation strategies promote equitable engagement. These measures, paired with evidence from studies that most students adapted well to independent learning, guide educators in creating inclusive, competency-based learning environments that nurture metacognitive development (31, 32).

Interestingly, a slightly greater proportion of female students rated self-directed learning as easy. This pattern suggests that, with appropriate support, female students may overcome initial challenges in less-structured environments and adapt to become effective self-regulated learners (33). Understanding these implications can inform the design of targeted support strategies to enhance learning outcomes for female students (34).

When aligned with clear instructional goals, visual collaboration tools like Miro can support student engagement and collaborative learning, suggesting that educators consider their integration into interactive clinical education. These findings advocate for broader adoption but should be interpreted in context, as they reflect self-reported perceptions rather than objective outcomes.

Future research should examine how visual collaboration tools impact objective learning outcomes, such as knowledge retention and clinical decision-making competence. Rigorous study designs and mixed-methods approaches are recommended to provide actionable evidence for educators considering these tools, supporting data-driven curricular decisions that align with observed student needs and experiences.

### 4.1 Limitations

This study’s single-institution design and small sample size may limit generalizability. Self-report questionnaires introduce response bias, including social desirability, non-response, and self-selection, which can affect representativeness. A cross-sectional design precludes assessment of long-term perceptions or outcomes related to Miro use. Further, no adjustments for multiple comparisons were applied. Although gender comparisons were included, other demographic factors, such as digital literacy or academic performance, were not assessed. The self-developed questionnaire, while clear to participants, has not been externally validated, which may limit its broader applicability.

The study relied solely on self-reported data, without objective measures of academic performance or knowledge retention. This approach introduces biases, such as social desirability and inaccurate self-assessment. As a result, findings are limited to perceived learning outcomes and may not represent actual educational effectiveness.

## 5 Conclusion

Our findings indicate that fifth-year medical students in pediatric medical education viewed the Miro app as a positive visual collaborative workspace. Students reported enhanced engagement, motivation, and clinical reasoning skills. Gender-based differences appeared in perceived value and some learning challenges, but both groups had a positive overall experience. These findings highlight the potential of structured digital collaboration tools to foster interactive, student-centered learning environments. However, conclusions regarding effectiveness or learning outcomes should be interpreted with caution, and further research incorporating objective measures is required to confirm these observations and evaluate long-term impact.

## Data Availability

The raw data supporting the conclusions of this article will be made available by the authors, without undue reservation.
